# Physiology and Scepticism

**Published:** 1861-03

**Authors:** 


### PHYSIOLOGISM AND SCEPTICISM.

### BRINTON'S LECTURE.—*Extract.*

So little of the truth, indeed, is there in the notion that the study of Physiology favors scepticism in religious matters, that the contrary statement might be maintained with considerably more semblance of accuracy. A subject which continually reminds us of our ignorance should scarcely make us vain; or which perpetually tells of what nevertheless we cannot observe with any of our senses, to doubt of the existence of a realm which at present lies altogether beyond their inquiry. Nay, more, if it were desired to minister to a mind diseased in this its most important part, it would be difficult to specify any better intellectual discipline than the study of Life and organization. If the sight of the stars in the sky could seem to the mind of the great Napoleon a scornful but complete answer to the sceptics who reasoned around him in his voyage to Egypt; and if he, in unconscious imitation, could draw from this spectacle the same lesson which David had done thousands of years before; surely the transcendent mystery which a drop of standing water reveals to modern observation—the throng of shapes, structures, instincts, and habits, which jostle each other in this microcosm—amount to no meaner teachings, and afford no less certain proof of a Creator. The honest doubter, I should rather say, may find in Physiology what, so far as his doubts are really intellectual, and not moral, must tend to shake and remove them. And any exception to this rule would almost justify the suspicion that his study of the subject was of that superficial and scanty character which, though it may, in the technical phrase of student life, grind men for the College or Hall, will most assuredly fit them for nothing useful in this world or the next.

The charge of Materialism is more difficult to deal with, mainly because of the sophistical sense in which the word has been used, and the odium it has therefore come to convey. To parody the sturdy Welshman's answer to the anti-Germanizing clergyman who casually held forth in his parish, "Here, sir, the great danger is of 'immaterialism.'" The only decently scientific hypotheses, in other words, which represent the material and its antagonist theory, are both of them, so far as I dare venture to judge, as perfectly compatible with religion as are the Books of Euclid. Indeed, I question whether they are not quite compatible with each other—whether the difference be not in great degree one of terms or words, rather than of ideas. One side, for example—well represented by one of the most able physiologists of the day, Dr. Carpenter—is inclined to assume the existence of a force or forces, so unlike anything we meet with in the inorganic creation—that is, so characteristic of life—as to be fitly named *vital.* These forces ceasing at death, allow the substance they previously tenanted and governed to fall back, as it were, into the domain of the ordinary laws of matter, in obedience to which they now decay and disperse into the world around them. The other side, which I will instance by the illustrious Valentin, regard Life and Organization as sustained in great measure by the same laws as those which, in different degrees and modes of working, operate in the inorganic world. Declining to separate physical and chemical phenomena of dead and living nature by a line of demarcation hitherto not definitely proved to exist, and confidently resting on the innumerable and undoubted facts which prove that such forces as heat, light, magnetism, chemical affinity, are every moment operating in the living body, it supposes that even the most peculiar and fleeting of these operations which we sum up as Life, are effected, in obedience to natural laws, by the use of forces everywhere present.

Of the theologian who should call this "Materialism," I would ask, "How does it affect revealed religion, or infringe our common creed?" Its supporters, in assuming that "the vital functions are the result of an infinitely wise plan of organization," do but modify, and indeed modify by exalting, our notions of the infinite wisdom they explicitly acknowledge. The materials, so to speak, they regard as fewer, simpler, more general, less heterogeneous, than had hitherto been imagined. Scientifically, they must only gain their position more fully by proving it more fully than they have yet done. But, theologically, if there be any difference, surely it is that the simpler the materials, the more unimaginable must be the skill and wisdom of the workmanship.

To balance between the two theories on physiological grounds is perhaps, less easy and conclusive. A critical intellect can, indeed, generally find something about the hypotheses more or less constrained and inexact. They never quite fit the limbs of growing science; and, to keep up this homely smile, Physiology, after limping uneasily about in them for a short time, soon treads them down at heel. The vitalist, for example, cannot question the share taken by inorganic forces in the ordinary functions; or the contrast between systemic and somatic death; between the death, for example, of the whole animal, and that death of its muscles which only comes on several hours after, and can be deferred or even removed by the injection of blood. The supporters of the opposite view, on the other hand, must be compelled to admit that we are still possibly ignorant of some of the forces which must essentially minister to life; and that, of these unknown forces, it is not impossible some may be more or less limited to organized or living matter. Each theory is thus more attractive according as you choose to foster your attention on the differences, or on the likenesses, of the forces concerned in the living and lifeless divisions of Nature. Each, however is only provisional; and if the latter may be regarded as any way preferable, it is only because it seems to be perhaps the more accurate and useful, precisely where it is the more modest, of the two; because it implies fewer consolations or excuses for our ignorance, and holds out larger incentives to our industry.—*London Lancet.*

---

PAIN.—*Superficial pains on both sides of the body, which are symmetrical, imply an origin or cause the seat of which is central or bilateral; unilateral pain implies a seat of origin which is one-sided, and as a rule, exists on the same side of the body as the pain.*

This is an important stand to take in endeavoring to unravel any obscure case through the medium of local pain. I must therefore repeat that in cases of symmetrical pains on the surface of the body, without the local manifestation of inflammation by an increased temperature of the parts, the cause must be central; and that if the pain be felt on one side only, the cause is only on one side, and it on the same side of the body as the pain.

Associated with disease in the lower cervical, or the lumbar or the dorsal vertebræ, the pains are almost always symmetrical, whilst in the diseases between the occiput and atlas, or between the atlas and the second vertebræ (the vertebra dentata) it often happens that the pains are unilateral, or one-sided.—*Hilton's Lecture.*

