# Erratum

**Published:** 1861-04

**Authors:** 


ERRATUM.—Upon page 144 heading in our March number, for PHYSIOLOGISM read PHYSIOLOGY. There are already *isms* enough in the world, without adding this barbar*ism.*

